# The Interplay of Host Microbiota and Parasitic Protozoans at Mucosal Interfaces: Implications for the Outcomes of Infections and Diseases

**DOI:** 10.1371/journal.pntd.0004176

**Published:** 2015-12-10

**Authors:** Ann-Katrein Bär, Niha Phukan, Jully Pinheiro, Augusto Simoes-Barbosa

**Affiliations:** 1 School of Biological Sciences, University of Auckland, Auckland, New Zealand; 2 Centre for Microbial Innovation, University of Auckland, Auckland, New Zealand; Universidad San Francisco de Quito, ECUADOR

## Abstract

Infections by parasitic protozoans are largely neglected, despite threatening millions of people, particularly in developing countries. With descriptions of the microbiota in humans, a new frontier of investigation is developing to decipher the complexity of host–parasite–microbiota relationships, instead of the classic reductionist approach, which considers host–parasite in isolation. Here, we review with specific examples the potential roles that the resident microbiota can play at mucosal interfaces in the transmission of parasitic protozoans and in the progress of infection and disease. Although the mechanisms underlying these relationships remain poorly understood, some examples provide compelling evidence that specific components of the microbiota can potentially alter the outcomes of parasitic infections and diseases in humans. Most findings suggest a protective role of the microbiota, which might lead to exploratory research comprising microbiota-based interventions to prevent and treat protozoal infections in the future. However, these infections are often accompanied by an unbalanced microbiota and, in some specific cases, apparently, these bacteria may contribute synergistically to disease progression. Taken together, these findings provide a different perspective on the ecological nature of protozoal infections. This review focuses attention on the importance of considering polymicrobial associations, i.e., parasitic protozoans and the host microbiota, for understanding these human infections in their natural microbial context.

## Introduction

**Fig 1:**
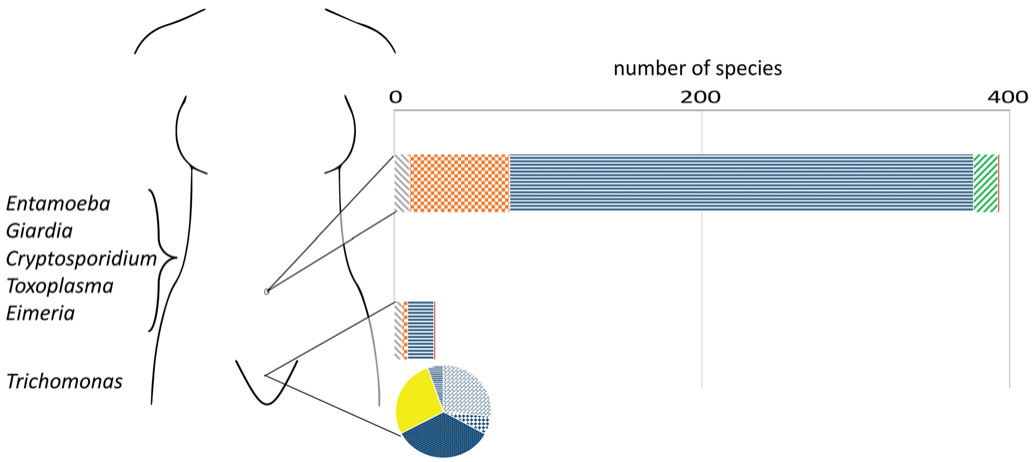
The microbiota of the human gut and vagina and the site-specific associated parasitic protozoans. The bar chart illustrates bacterial diversity at species level, grouped by phylum, found in the gut (top bar) [4] and vagina (bottom bar) [9]. Bacterial phyla found in the gut are, from left to right: Actinobacteria, Bacteroidetes, Firmicutes, Proteobacteria, and the least diverse group of Fusobacteria. Bacterial phyla found in the vagina are, from left to right: Actinobacteria, Bacteroidetes, Firmicutes, and Fusobacteria. Despite showing higher species diversity in comparison to the vagina, the relative abundance of bacterial species in the gut varies greatly among individuals [5]. In the vagina, however, the microbiome can be categorized into five microbial communities as illustrated in the pie chart. Four of these are dominated by a single species of *Lactobacillus* (phylum Firmicutes). These species, coloured in blue, are shown on the pie chart from top to bottom in an anticlockwise direction: *L*. *jensenii*, *L*. *crispatus*, *L*. *gasseri*, and *L*. *iners*. A fifth community, coloured in yellow, is composed of a highly diverse polymicrobial community containing mostly anaerobic bacteria such as *Prevotella bivia*, *Atopobium vaginae*, *Gardnerella vaginalis*, *Megasphaera* sp., and *Sneathia* sp., [9]. Parasitic protozoans of human gut and vagina are listed on the left. The only vaginal protozoan of humans is the extracellular parasite *Trichomonas vaginalis*. Except for the extracellular parasite *Entamoeba histolytica* and the intracellular parasite *Toxoplasma gondii*, these protozoans are site-restricted and cause self-limiting infections. The interplay of these parasites with the human microbiota is discussed in this review.

Parasitic protozoans contribute significantly to the burden of infectious diseases worldwide and represent a major public health problem. Most of the people affected by these infections live in developing countries, and these diseases remain neglected, receiving little funding and intervention. Parasitic protozoans that infect and colonise or infect and transit human mucosas are extremely prevalent; examples of these are illustrated in Fig 1. While *Toxoplasma gondii* is one of the most prevalent human infections acquired via ingestion, *Trichomonas vaginalis* is the most prevalent sexually transmitted infection of non-viral cause worldwide [1,2].

The mucosal surfaces of the human body are colonised by stable communities of microorganisms, mostly bacteria, which are collectively known as the human mucosal microbiota. The development of this microbiota starts at birth and evolves naturally, and its composition is influenced by the environment significantly. A stable bacterial consortium becomes established early in life, in which these species act collectively to modify and to metabolize substrates optimally in particular niches of the host’s mucosa. Some of these bacteria benefit the host in aspects of nutrition, immune development, and protection against pathogens [3]. Parasitic protozoans that infect the mucosal surfaces can potentially interact with these local bacterial residents.

At least 400 different species of bacteria are found in the gastrointestinal tract of humans (as revealed by faecal sampling and metagenomics [4,5]), which is a common site of infection by parasitic protozoans (Fig 1). Despite this relatively high number of distinct bacterial taxa, they belong to a relatively small number of phyla [6–8]. Bacteroidetes and Firmicutes are the most abundant taxa of this microbiota, but their relative abundance varies greatly among individuals [5].

The human vagina is also colonised by site-specific bacterial communities as revealed by metagenomics of vaginal swabs [9]. In general, the microbiota of the human vagina is at least ten times less diverse than the one described in the gut by faecal sampling and metagenomics (Fig 1). In addition, the vaginal microbiota can be categorized into five distinct community types. Lactobacilli (Firmicutes) comprise >95% of total vaginal bacteria in four out of the five community types. This microbial profile, where lactobacilli are the dominant species, is found in about 75% of women. On the other hand, a distinctive community having low proportions of lactobacilli and dominated by various species of anaerobic bacteria is found in the other ~25% of women (Fig 1) [9]. The gut and vagina are sites of infection for a number of parasitic protozoans of medical importance (Fig 1).

Various reviews summarize the important contribution of the microbiota to normal human physiology [10]. The present review focuses on the role of the microbiota in influencing the outcomes of protozoal infections and diseases in humans. In the following sections, specific examples will highlight the protective roles of bacterial components of this microbiota against parasitic protozoans at gut and vaginal mucosal interfaces. On the other hand, other bacteria apparently trigger specific changes in the behaviour of host and parasite, which could potentially aid invasion, infection, and disease. Parasite and host interactions are very often examined in isolation. These findings suggest reconsiderating the impact of the microbiota in these interactions. A better understanding of this subject might provide alternatives for prevention and treatment of these prevalent human infections in the future.

## The Interplay of Human Microbiota and Parasitic Protozoans

Infections by parasitic protozoans are generally associated with changes in the structure and composition of the commensal bacteria. By separately examining intracellular and extracellular forms of parasitic protozoans during their development in humans, this section of the review reveals intimate relationships between the host, the native bacterial microbiota, and protozoans, which impact the outcomes of many of these medically important infections.

### Intracellular parasitic protozoans

Parasitic protozoans that are mainly intracellular are specialized to evade and to manipulate the immune response. For this reason, these infections frequently lead to immunopathologies in which the progress of the disease will depend on the type and level of the immune response. In this scenario, the potential benefit of the human microbiota is to provide a more effective immune response against infections [3]. Although such correlations have been found, the effects of the microbiota on host immune response to protozoal infections have only been examined in a few examples, as discussed below.

Apicomplexans (phylum Apicomplexa) are obligatory intracellular parasites exhibiting an unusual type of motility and associated organelles necessary for host cell invasion. They also harbour a unique plastid-type of organelle (the apicoplast) and display asexual and sexual modes of reproduction with formation of spores. Coccidians are apicomplexan parasites that utilise intestinal epithelial cells of vertebrates (including humans) as a transient or final habitat. The impact of gut microbes on the infection of the coccidian parasites *Cryptosporidium* and *Eimeria* is variable and unclear. In *Cryptosporidium parvum*, an opportunistic intestinal coccidian parasite of humans, germ-free and immunodeficient mice develop heavy infections after a few weeks, which is in sharp contrast to the same immunodeficient mice harbouring a normal microbiota [11]. On the other hand, some species of *Eimeria* (intestinal coccidian parasites of many vertebrates) fail to infect germ-free animals as compared to conventionally reared and microbial-colonized animals [12,13].

**Fig 2:**
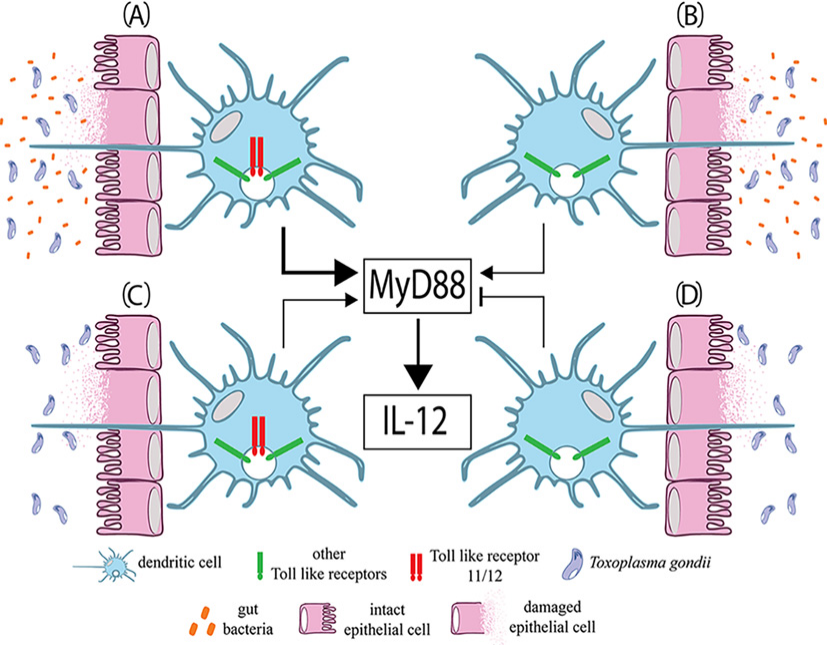
Initiation of mucosal innate immune response via dendritic cells against *Toxoplasma gondii* infection in mice. The toll-like receptor (TLR)-adaptor protein MyD88 is a key element to the protective response based on production of IL-12. Secretion of IL-12 will trigger an effective cellular-based immune response with production of INF-γ and activation of a Th1 T lymphocyte profile. (A) This innate response is mainly dependent on TLR11, which forms endolysosomal dimers with TLR12 that recognize profilin from *T*. *gondii*. This recognition is central to mucosal immunity triggering production of IL-12. (B) In the absence of TLR11, however, this response is still minimally and sufficiently compensated by indirect stimulation provided by the gut microbial commensals via TLR2, TLR4, and TLR9 [44]. In this case, infection-induced cell destruction and intestinal dysbiosis apparently trigger loss of tolerance to gut commensals. When the gut microbiota is severely reduced by prolonged antibiotic treatment, the following observations can be made: (C) Wild-type mice expressing TLR11 exhibit a reduced but not abolished IL-12 response. These animals can still build up Th1 immunity. (D) TLR11-knockout mice are unable to mount IL-12 responses against this parasite, and Th1 immunity is severely impaired. In conclusion, gut commensals serve as natural molecular adjuvants during *T*. *gondii* infection.

*T*. *gondii* is a model organism among apicomplexan parasites. It causes toxoplasmosis, which, despite being clinically asymptomatic in a vast majority of cases, is the most prevalent human parasitic infection worldwide [14]. The very successful spread of *T*. *gondii* in human populations relies on its unusual biology and the way that humans respond to this infection, as described below. Like no other intracellular parasitic protozoan, *T*. *gondii* is extremely host generalist, being able to invade and multiply in any nucleated cell of any warm-blooded animal. In addition, *T*. *gondii* is easily transmitted to humans by ingestion of either environmentally-resistant spores released from cat faeces or pseudocysts present in infected meat from various sources. Lastly, but no less important, a protective and Th1-polarized adaptive immune response normally develops in immunocompetent individuals [15,16], and human infections become clinically silent in most cases. Gut commensal bacteria might largely contribute to the development of this protective immune response and the associated pathology [17], as discussed below (Fig 2).

When *T*. *gondii* reaches the gut of an individual with a native and functional microbiota, an immune response is initiated at the level of the intestinal mucosa by activation of toll-like receptors (TLRs) in dendritic cells (DCs) (Fig 2). In vitro studies point to the involvement of various TLRs such as TLR2, TLR3, TLR4, TLR7, and TLR9 in response to *T*. *gondii* infection [18]. However, experiments in mice reveal that recognition of *T*. *gondii* profilin by the dimer TLR11/12 has a key role in the immune response against oral infection (Fig 2A and 2C) [18,19]. Profilin is the major parasite ligand that activates TLR11. Because profilin is an intracellular protein, it is either released by an unknown mechanism or taken up by DCs when they phagocytose dead parasites and debris. This recognition activates the transcription factor interferon (INF) regulatory factor 8, leading to the production of interleukin 12 (IL-12) [20,21]. IL-12 promotes a cellular-based immunity with production of INF-γ from natural killer cells and differentiation of Th1 T lymphocytes. As described below, these cells and molecules have a role in immunopathogenesis of *T*. *gondii* infections and are also required to initiate the adaptive phase of the immune response [18].

This IL-12 response in mice depends on the TLR-adaptor MyD88 and the myeloid differentiation factor 88 [22]. This molecule has a central role in the immune response against *T*. *gondii* oral infection (Fig 2). In MyD88 genetically ablated mice, IL-12 response is non-existent, and these knockout mice perish within two weeks after oral infection [23]. TLR-adaptor molecules, such as MyD88, can respond to many types of TLRs. MyD88 initiates various intracellular signalling pathways, but the resulting balance of co-stimulatory molecules depends on the context of the TLR ligands [24]. In the context of TLR11/12, it triggers the release of IFN-γ via the NF-κB pathway, which stimulates macrophages and CD8α^-^ DCs “priming” the immune response [18].

Importantly, *TLR11* ^-/-^ mice are not completely impaired to respond to *T*. *gondii* as long as gut commensal bacteria are present (Fig 2B and 2D). In the presence of gut commensals (Fig 2B), partial resistance is retained with a decreased but existent IL-12 response that is sufficient to produce similar amounts of INF-γ as compared to wild-type mice. Therefore, *TLR11* ^-/-^ mice survive the acute phase of infection. When *TLR11* ^-/-^ mice are treated with antibiotics in order to eliminate gut commensal bacteria, this remaining protective IL-12 response is lost (Fig 2D). This protective response can be rescued with oral administration of bacterial lipopolysaccharide (LPS) [17]. Furthermore, TLR-knockout mice indicate the involvement of TLR2, TLR4, and TLR9 in the ability of mounting a minimally sufficient INF-γ response to the parasite. These TLRs are all MyD88-dependent but do not respond to *T*. *gondii* infection per se. LPS, for instance, is a bacterial agonist of TLR4. Therefore, gut commensal bacteria function as a natural molecular adjuvant during oral infection by *T*. *gondii*, providing an indirect stimulation of mucosal DCs. The natural community of gut microbes are apparently necessary since *Bacteroides*, an abundant genus of gut bacterium, cannot provide this stimulation alone [17].

Epithelial damage, resulting from initial replication of *T*. *gondii* inside intestinal epithelial cells, should allow translocation of lumen bacteria to the lamina propria. In addition, *T*. *gondii* oral infection leads to intestinal dysbiosis (i.e., bacterial imbalance) with deregulation or loss of Paneth cells [19]. These specialized intestinal epithelial cells produce antimicrobial peptides, which are important for maintaining bacterial homeostasis in the gut. *T*. *gondii-*induced dysbiosis is characterized by a decrease of gut bacterial diversity with a shift toward gram-negative bacteria, a key source of LPS [19]. Commensal-derived flagellin, the ubiquitous and main protein of a bacterial flagellum, could also be a source of indirect stimulation of T lymphocytes [25]. Altogether, DCs apparently trigger a loss of immunological tolerance against the gut bacterial flora.

This indirect immunological stimulation might be important to the immunopathology of *T*. *gondii* infection in mice. In the absence of TLR11, gut bacteria not only provide a sufficient Th1 response but also preclude the development of the immunopathology of toxoplasmosis in orally infected mice [17]. Humans lack a functional TLR11, which is found as a pseudogene, and an equivalent TLR sensor for profilin has not been found. In addition, it is unclear if TLR12 alone can accomplish the role of TLR11 [18]. The involvement of other TLRs and a TLR-independent mechanism for parasite recognition in human oral infections are not yet clear [18]. Meanwhile, it is tempting to speculate an interesting evolutionary scenario involving host, parasite, and bacterial microbiota. The co-evolution of *T*. *gondii*, humans, and their gut microbiota has had major impact on each other, favouring survival of both host and parasite. The co-evolution of this triad has resulted in preservation of the host, which is characterized by a general lack of life-threatening symptoms in infected humans, and has contributed to the successful spread of *T*. *gondii* in human populations worldwide.

### Extracellular parasitic protozoans

Some parasitic protozoans of humans are exclusively extracellular. Examples are *Entamoeba histolytica*, *Giardia lamblia*, and *Trichomonas vaginalis*. Living outside of human cells and tissues, these parasites should physically interact with the native mucosal microbiota. By maintaining intimate and cooperative relationships among themselves and with the host, the native bacterial microbiota of the human gut and vaginal mucosa might represent a natural barrier to pathogen invasion. The interactions between extracellular parasitic protozoans and the human mucosal microbiota, their effects, and their mechanisms, are presented in this section.

*E*. *histolytica* is a human-specific extracellular parasite found in the lumen of the large intestine, the site that contains the largest bacterial population density in humans. The severity of ameobiasis is linked to the ability of this parasite to leave the intestinal lumen and to destroy the intestinal mucosa, promoting haemorrhagic dysentery and disseminating to other organs, a severe condition known as invasive or extraintestinal ameobiasis [26]. In early studies, the virulence of *E*. *histolytica* was shown to be significantly enhanced by intestinal bacteria in microbiota-controlled animal models [27,28]. Further insights into this microbial relationship have been gained under in vitro and laboratory-controlled experimental conditions [29,30].

Modulation of *E*. *histolytica* virulence by intestinal bacteria varies with time, species or strains of bacteria, and strains of the parasite [31–35]. The bacterial strain of *Escherichia coli* serotype O55 binds strongly to the surface Gal/GalNAc lectin of *E*. *histolytica* because of its natural surface carbohydrate composition rich in galactose and N-acetyl galactosamine. Recognition of *E*. *coli* O55 by *E*. *histolytica* via Gal/GalNAc, a central virulence factor of this amoeba, enhances its virulence in the first hour probably as a result of the activation of downstream pathways triggered by Gal/GalNAc recognition. However, in the course of the first month of monoaxenic cultivation, there is a significant reduction in virulence, which is restored after three months of further co-cultivation. This influence on the amoeba’s virulence was observed at different levels, such as regulation of Gal/GalNAc expression, phagocytosis, proteolysis, adhesion, and cytotoxicity. The interaction between this parasite and endogenous bacteria causes substantial changes in the amoeba’s gene expression, suggesting that some of these bacteria may also have a nutritional role that supports amoebic growth [31,34].

Other intestinal and non-intestinal pathogenic bacteria including *E*. *coli*, *Shigella dysenteriae*, and *Staphylococcus aureus* also promote substantial changes at both *E*. *histolytica* virulence and host response. Significant enhancement of virulence properties of *E*. *histolytica* was observed with an increase of Gal/GalNAc expression, proteolysis, adhesion, and cytotoxicity. Notably, this enhancement on the amoeba’s virulence was only seen with these pathogenic bacteria and not with non-pathogenic commensal *E*. *coli*. In addition, *Entamoeba dispar* (an intestinal non-pathogenic amoeba closely related to *E*. *histolytica*) was unable to promote these effects even when combined with the same pathogenic bacteria. Importantly, these pathogenic bacteria also altered the host response with respect to epithelial barrier function, chemoattraction of neutrophils, and inflammatory response [32,35]. Altogether, the aforementioned studies suggest that synergistic effects of some intestinal bacteria on the host and parasite responses may provide an environment more permissible for parasite invasion, which could contribute to disease development. Because the human gut microbiome is a complex system involving at least hundreds of bacterial species, these initial observations may still represent an oversimplification of what happens in intestinal infections by *E*. *histolytica* under natural conditions.

*Giardia lamblia* is another extracellular parasitic protozoan found in the lumen of the human intestine. However, in contrast to *E*. *histolytica*, *G*. *lamblia* is not invasive and resides in the small intestine, where microorganisms are not as abundant [36]. Individuals with *G*. *lamblia* infection (i.e., giardiasis) display alterations in the bacterial composition of the upper digestive tract [37,38]. The intestinal bacterial microbiota may potentially influence the outcomes of giardiasis, but it is unclear if these microbial alterations are cause or effect. Although lactobacilli can inhibit *G*. *lamblia* proliferation in vitro [39] or could help as a probiotic intervention in animal models [40–42], it is unclear if these bacteria are autochthonous or indigenous inhabitants of the site of *G*. *lamblia* infection in humans. An intriguing report has shown that natural resistance to *G*. *lamblia* infection between mice of similar genetic background but originating from different breeders can be transferred between these animals solely by housing animals together for few weeks [43]. Although antibiotic treatments and partial characterization of these microbial communities suggest a protective role of the gut microbiota, the mechanisms underlying resistance to *G*. *lamblia* infection in these animals remain elusive. The existing evidence does not yet clarify the role of the gut microbiota in giardiasis.

*T*. *vaginalis* is the only parasitic protozoan of the human genital tract. It causes trichomoniasis, the most common sexually transmitted infection of non-viral etiology worldwide [2]. In the vagina of women of childbearing age, this extracellular parasite invades the natural habitat of a dense and resilient community of bacteria. The vaginal microbiome of 396 asymptomatic North American women was recently characterized into five community state types (Fig 1, pie chart). Four of these are dominated by different species of *Lactobacillus*—*L*. *iners*, *L*. *crispatus*, *L*. *gasseri*, and *L*. *jensenii*—with a relatively high (and almost exclusive) taxon abundance. Except for *L*. *iners*, lactobacilli are poorly represented in the fifth community type, which is predominantly composed of anaerobic bacteria such as *Atopobium vaginae*, *Prevotella bivia*, *Megasphaera* sp., *Sneathia* sp., and *Gardnerella vaginalis* [9]. Eleven cases of asymptomatic *T*. *vaginalis* infections (i.e., 2.8%) were found in this group of women [44]. Observing the distribution of *T*. *vaginalis* infections amongst these bacterial community types, two important observations can be made. Firstly, there was a low abundance of lactobacilli in the vagina of 73% of the *T*. *vaginalis*- infected women (8/11), which is in agreement with previous clinical reports [45]. The exception, once again, was *L*. *iners*, which was found in a taxon abundance of >80% in the vagina of 18% of *T*. *vaginalis-*infected women (2/11). Secondly, those *T*. *vaginalis* infections lacking lactobacilli (8/11) were associated with the fifth community type, containing mostly anaerobic bacteria. Such microbial shift, with exclusion of lactobacilli and higher prevalence of these anaerobes, is typically associated with a common disease condition known as bacterial vaginosis (BV). *L*. *iners* has also been linked to this condition [46].

A note of caution, however, should be added. Firstly, this is a representation of an asymptomatic population displaying a relatively small number of *T*. *vaginalis* infections (11/396). Secondly, BV is a common condition in African-American populations [47], and this cohort was highly represented in this study [9,44]. Additional metagenomics studies comprising various ethnic groups may be needed to confirm unequivocally the existence of a strict association between *T*. *vaginalis* infection and a specific vaginal microbial community. If such a microbial association exists, two hypotheses might be drawn: (i) Lactobacilli and *T*. *vaginalis* are competitors interacting antagonistically in their natural environment; (ii) BV bacteria and *T*. *vaginalis* interact cooperatively, and the disease trichomoniasis might result from the interactions of *T*. *vaginalis* with one or more types of BV bacteria.

Using polymicrobial infection models in tissue culture, two recent studies give support to the hypotheses above [48,49]. Firstly, *T*. *vaginalis* was found to reduce numbers of *Lactobacillus acidophilus*, *L*. *jensenii*, and *L*. *crispatus* [48]. Secondly, *T*. *vaginalis* adhesion to human cells (a key aspect of its virulence) was significantly inhibited by *L*. *gasseri* in strain-specific and contact-dependent manners [49]. Finally, two common BV-associated bacteria, *Atopobium* and *Gardnerella*, were found to cause synergistic enhancement of *T*. *vaginalis*-induced chemokines [48]. In addition, clinical isolates of *T*. *vaginalis* often harbour mycoplasmas. *Mycoplasma hominis*, in particular, is frequently associated with enhanced inflammation during trichomoniasis [50]. *M*. *hominis* was shown to upregulate proinflammatory responses of human monocytes to *T*. *vaginalis* infections in vitro in a synergistic way [51].

Taken together, these recent studies suggest that microbial associations of this nature might influence significantly the outcomes of trichomoniasis by favouring both the growth of associated pathogenic bacteria and creating an inflammatory environment more permissible for disease development.

## Discussion and Perspectives

The number and diversity of microbial cells living on the mucosal surface of humans should impact infections by parasitic protozoans that transit or reside on mucosal surfaces. Commensal microorganisms contribute a large repertoire of unique genes to their hosts, whose products are likely to impact the functioning of the host and invading parasites. This review presents evidence that the microbiota of humans can significantly alter the outcomes of various protozoal infections. However, the cellular and molecular mechanisms underlying these mutual responses are yet to be deciphered in most cases.

The existence of both synergistic and antagonistic interactions between hosts, parasites, and microbiota, as presented in this review, expands our understanding of the ecological nature of parasitic infections. Evidence for synergistic associations between mucosal bacteria and protozoans was seen in the gut with *E*. *histolytica* and in the vagina with *T*. *vaginalis*. Importantly, the synergistic microbial interactions might potentially facilitate infection and aid progression of these parasitic diseases in humans. While these protozoan infections are frequently associated with an unbalanced or dysbiotic microbiota, this observation questions the monoetiological origin of these parasitic diseases. A broader spectrum of these relationships (host–parasite–microbiota instead of simply host–parasite) must be considered for a proper understanding of these diseases in natural situations in which human mucosal surfaces are fully colonised by a great diversity of microorganisms.

In the case of antagonistic interactions between bacterial microbiota and parasites, the potential use of this knowledge to develop better treatments for protozoal infections in humans is an exciting topic. Firstly, if these antagonistic interactions between parasites and human mucosal bacteria prove to be detrimental for the parasites, the off-target effects of antiparasitic drugs on the host microbiota and/or replenishment of the normal microbiota after drug treatment need to be considered.

Secondly, bacteria are genetically adaptable. Deciphering the genetic traits underlying this host-protective response against specific protozoal infections will allow producing laboratorial strains that might prove useful as alternative therapies. These improved strains could potentially help the prevention or the treatment of parasitic infections. This is relevant particularly in cases of immunosuppression or in cases where conventional treatment should be minimized or avoided (e.g., pregnancy, drug toxicity, and drug resistance). Therapeutic trials by modifying the gut microbial ecosystem with pre-, pro-, and symbiotics, and even genetically modified bacteria, have been attempted [52], including treatment against parasites of medical importance [53].

Finally, and as a note of caution, most research has taken a classic reductionist approach (i.e., host–parasite), whereby these interactions have been studied without consideration of the surrounding microbial context (i.e., host–parasite–microbiota). This review indicates that the microbiota might have an important impact on protozoal infections in humans. Our knowledge about these diseases will improve largely once the interactions between host–parasite–microbiota and their outcomes are elucidated. This knowledge might lead to the development of microbiota-based interventions in humans to control parasitic infections in the future.

**Top Five Papers**

1. Benson A, Pifer R, Behrendt CL, Hooper LV, Yarovinsky F. Gut commensal bacteria direct a protective immune response against Toxoplasma gondii. Cell Host Microbe. 2009; 6(2):187–196. doi: 10.1016/j.chom.2009.06.005.
2. Yarovinsky F. Innate immunity to Toxoplasma gondii infection. Nat Rev 415 Immunol. 2014 Feb;14(2):109–21.
3. Galván-Moroyoqui JM, Del Carmen Domínguez-Robles M, Meza I. Pathogenic bacteria prime the induction of Toll-like receptor signalling in human colonic cells by the Gal/GalNAc lectin Carbohydrate Recognition Domain of Entamoeba histolytica. Int J Parasitol. 2011 Aug 15;41(10):1101–12.
4. Fichorova RN, Buck OR, Yamamoto HS, Fashemi T, Dawood HY, Fashemi B et al. The villain team-up or how Trichomonas vaginalis and bacterial vaginosis alter innate immunity in concert. Sex Transm Infect. 2013; 89(6):460–466. doi: 10.1136/sextrans-2013-051052.
5. Phukan N, Parsamand T, Brooks AE, Nguyen TN, Simoes-Barbosa A. The adherence of Trichomonas vaginalis to host ectocervical cells is influenced by lactobacilli. Sex Transm Infect. 2013; 89(6):455–459. doi: 10.1136/sextrans-2013-051039.

**Key Learning Points**

- Parasitic protozoans do not live in isolation but interact with microbial communities that naturally colonise the surfaces of humans (i.e., microbiota). The nature of these interactions (antagonism or synergism) can significantly change the outcomes of these infections.
- Dysbiosis (i.e., an unbalanced microbiota) is frequently associated with protozoal infections in humans. Although the cause–effect is unclear in most situations, many correlations have been attributed to justify the protective effect of the normal microbiota against protozoal infections.
- The effect of the gut microbiota in the immunopathogenesis of toxoplasmosis has been described in mice, and the mechanism is partially understood. This model places the microbiota as molecular adjuvants at the gut level helping protect the host. A parallel scenario may explain the high prevalence and asymptomatology of this infection in humans.
- In the extracellular parasites *Entamoeba histolytica* and *Trichomonas vaginalis*, some local bacteria trigger changes on host and parasite responses that may increase severity of disease. The mechanisms are not completely understood yet.
- Understanding the interplay of host–microbiota–parasite will advance our knowledge about these diseases and may lead to development of novel treatments against these infections based on microbiota intervention.
